# Supplementary material for: A natural WNT signaling variant potently synergizes with Cdkn2ab loss in skin carcinogenesis
Source: Nat Commun. 2019 Mar 29;10:1425. doi: 10.1038/s41467-019-09321-8 (PMC6441055; doi:10.1038/s41467-019-09321-8)
Supplement: Supplementary file 1 — Supplementary Information [file 41467_2019_9321_MOESM1_ESM.pdf]

Supplementary information

Manuscript:

A natural WNT signaling variant potently synergizes with *Cdkn2ab* loss in skin carcinogenesis

Krimpenfort et al.

Suppl. figure 1

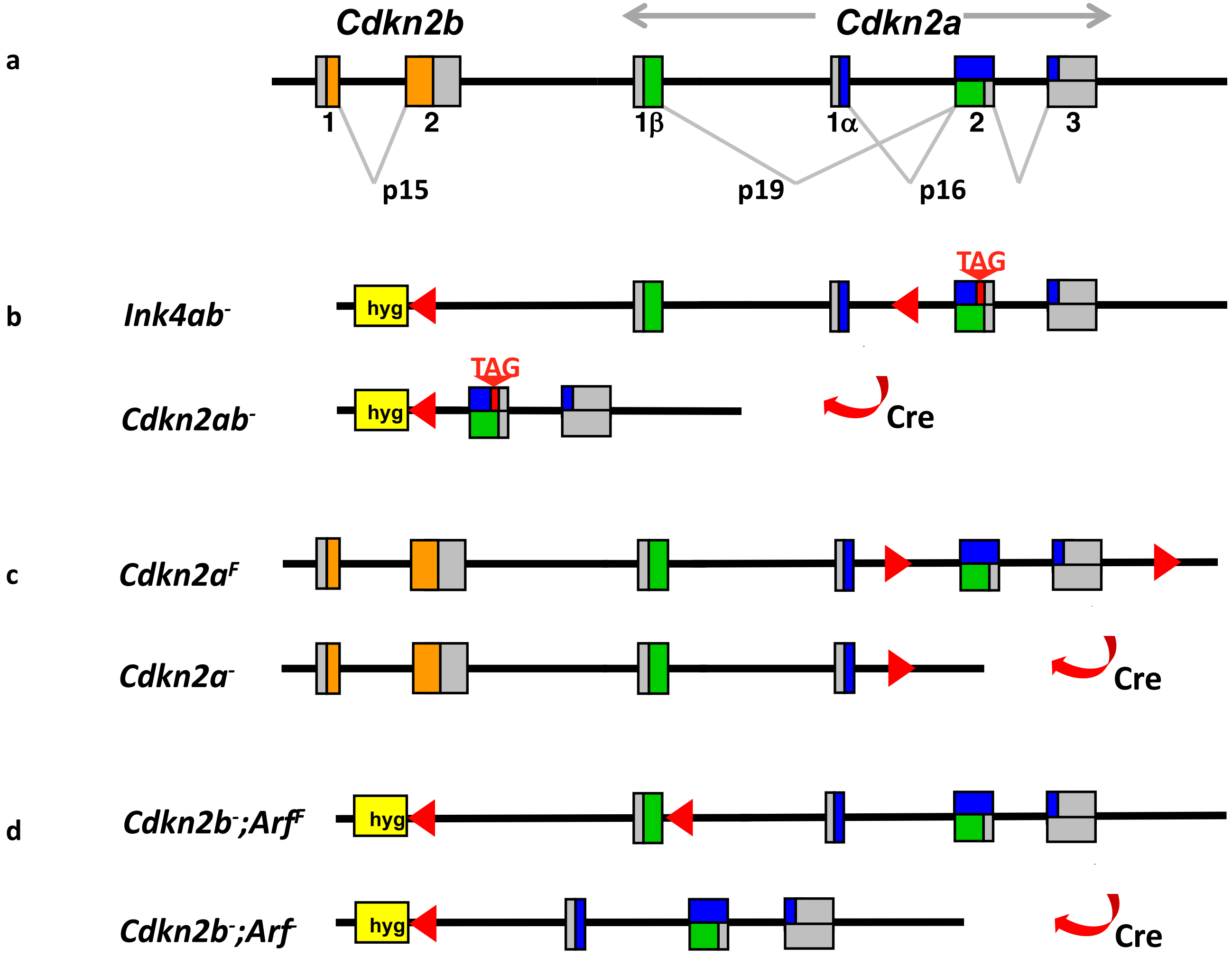

Suppl. figure 1) *Cdkn2ab* mutant alleles used in this study. a) Schematic of *Cdkn2ab* locus. **b)** The *Ink4ab*$^-$ allele (knockout for p15^{Ink4b} and p16^{Ink4a}) (ref 2) enabling the Cre mediated derivation of the *Cdkn2ab*$^-$ allele (knockout for p15^{Ink4b}, p19^{Arf} and p16^{Ink4a}). **c)** The conditional *Cdkn2a*^{F} allele enabling the Cre mediated derivation of the *Cdkn2a*$^-$ (knockout for p16^{Ink4a} and p19^{Arf}) (ref 9). **d)** The *Cdkn2b*$^{-/-}$*;Arf*$^{F/F}$ mutant allele (knockout for p15^{Ink4b} and conditional knockout for p19^{Arf}) enabling the Cre mediated derivation of the *Cdkn2b*$^-$*;Arf*$^-$ allele (knockout for p15^{Ink4b} and knockout for p19^{Arf}). The location of LoxP sites are indicated by red triangles.

Suppl. figure 2

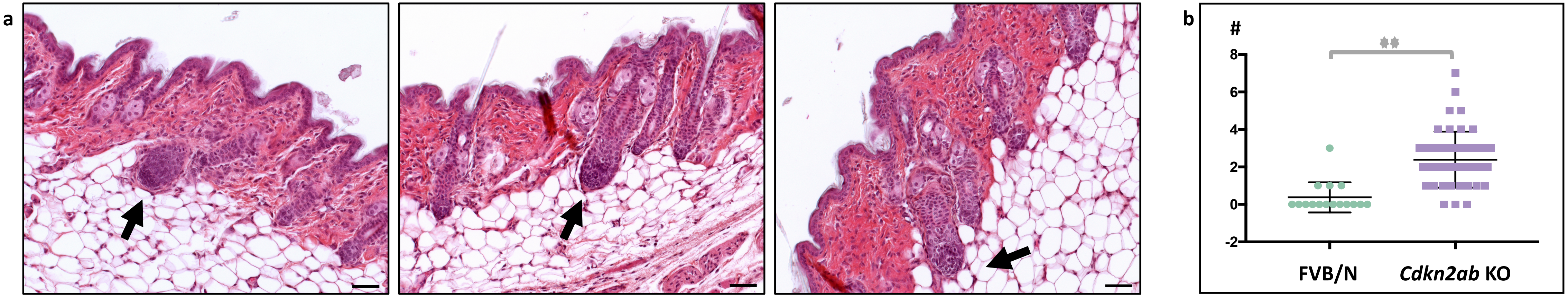

Supplementary figure 2) *Cdkn2ab*$^{-/-}$ mice show hypertrophic hair follicles in telogen phase skin. a, H&E staining of representative skin (arrow, scale bar: 20 μm). **b,** Quantification of number (Y-axis) of hyperproliferative hair follicles per section in wild-type FVB/N vs *Cdkn2ab* mutant mice.

Statistical analysis (Unpaired t test): **: p< 0.01.

Suppl. figure 3

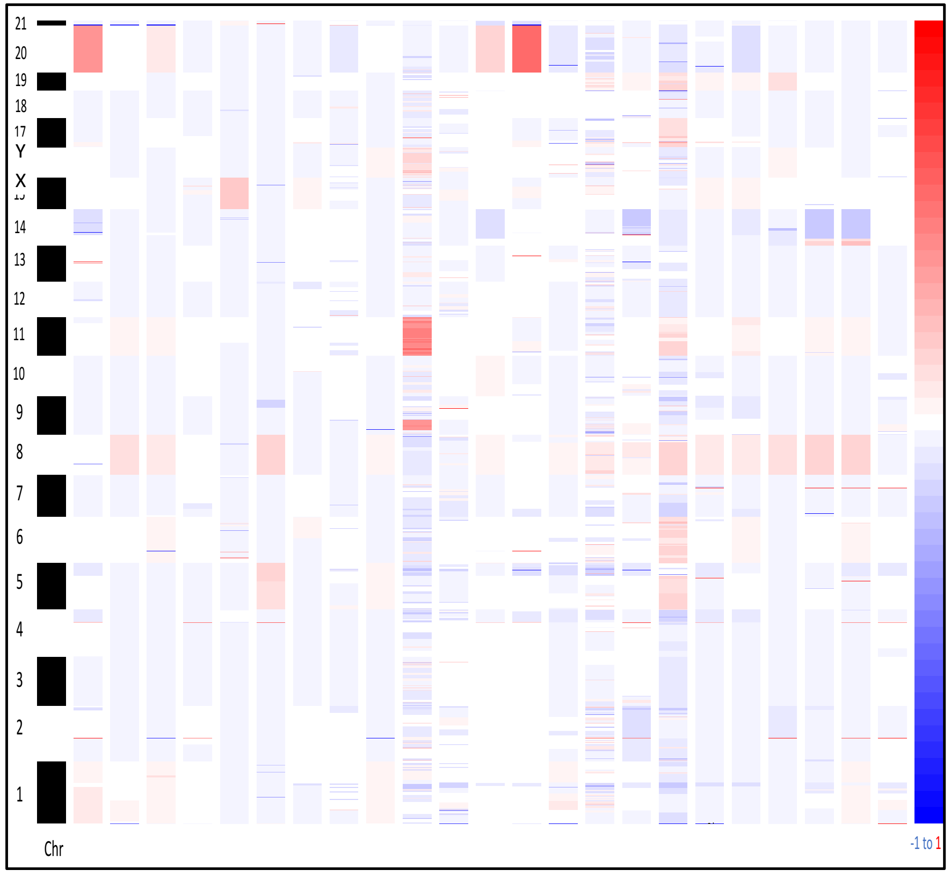

Supplementary figure 3) Overview of somatic copy number changes in 23 mouse tumours. For each sample (column, X-axis), copy number changes are indicated by color (blue indicates loss; red, gain) from chromosomes 1 to 19, X and Y (Y-axis).

Suppl. figure 4

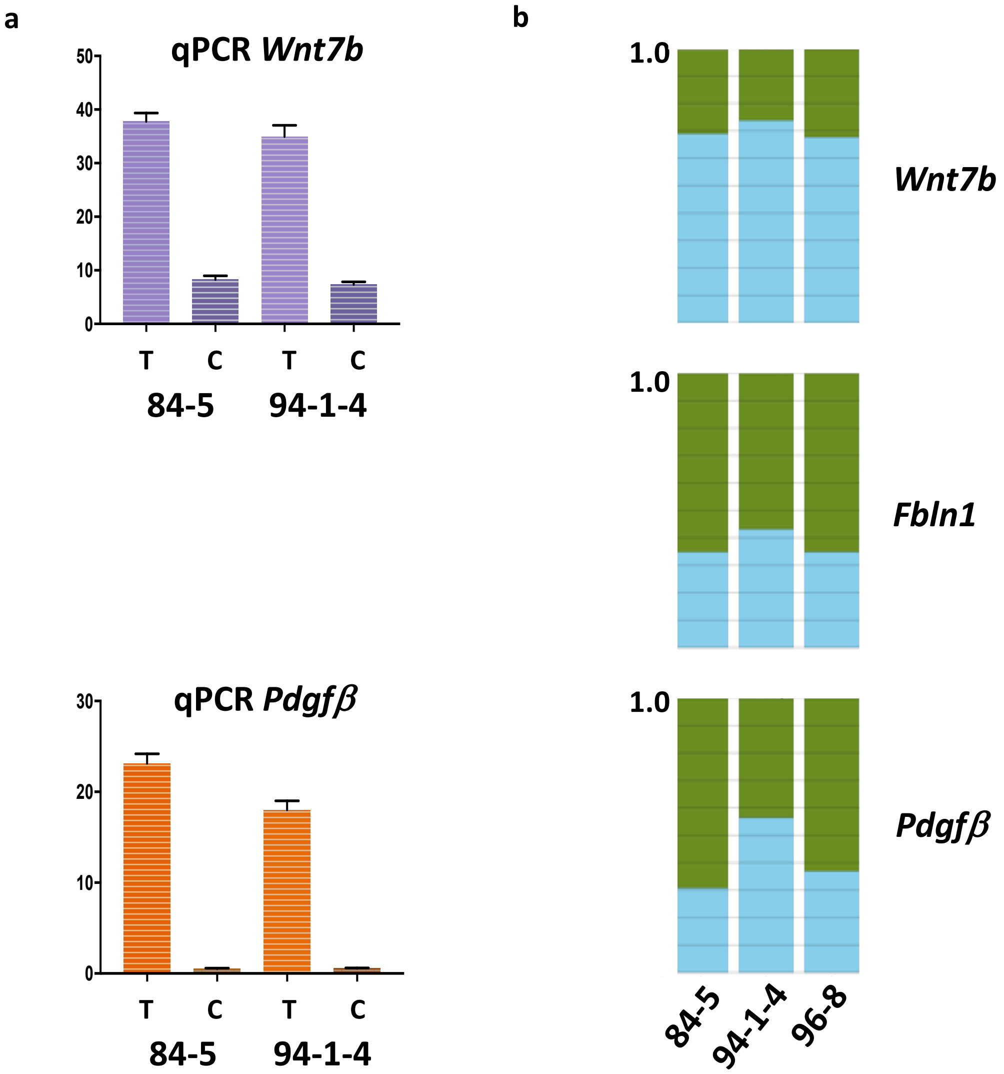

Suppl. figure 4) **a,** mRNA analysis (qPCR) Of Wnt7b and Pdgfβ in primary tumours (T) and cell lines derived (C) there of. **b,** Fraction of FVB/N allele derived transcripts (green) and 129P2 allele derived transcripts (blue) of *Wnt7b*, *Fbln1* and *Pdgfβ* in 3 cell lines derived from primary skin tumours.

Suppl. figure 5

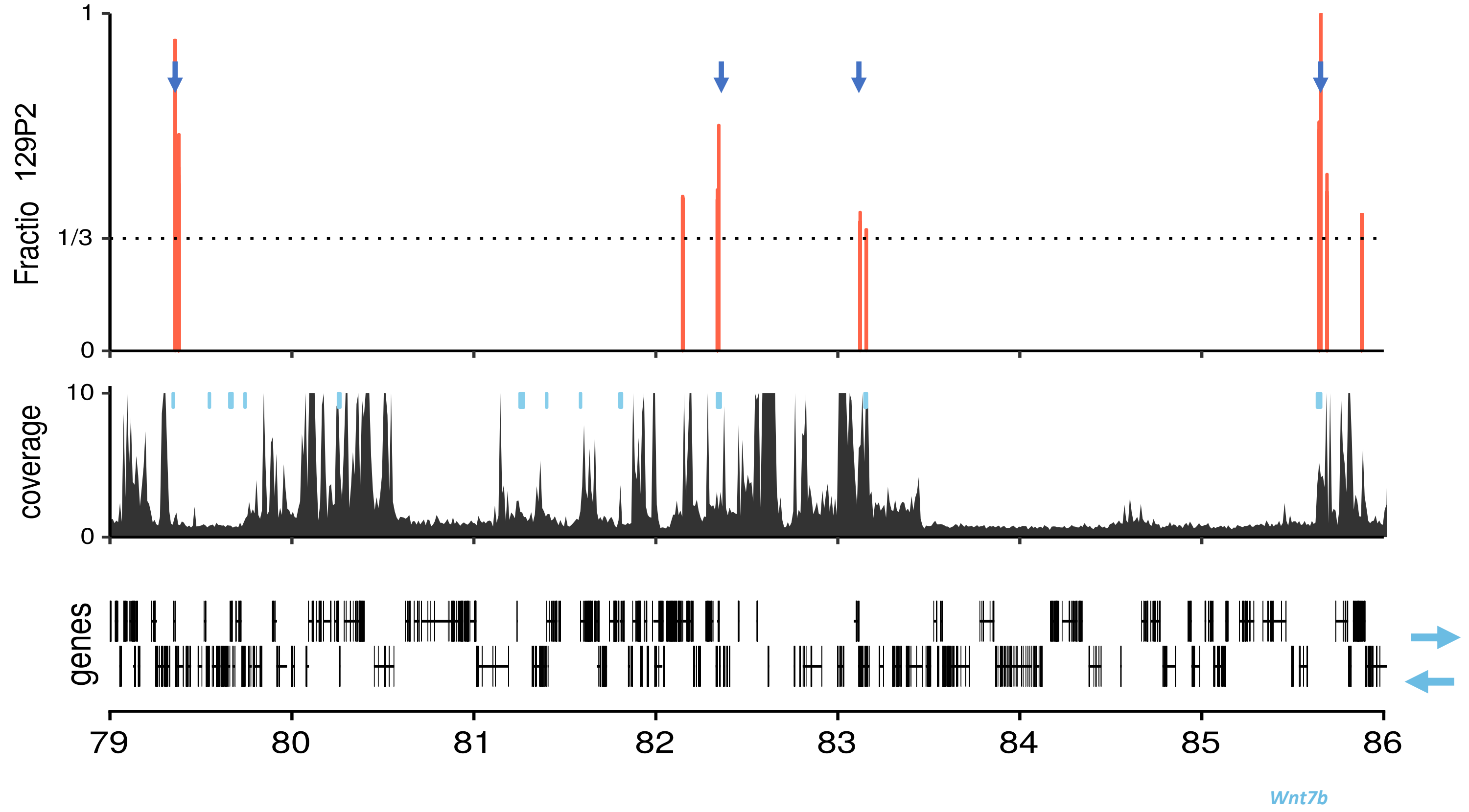

Suppl. figure 5) Window of region of chr15:79-86Mb with significant enrichment of the 129P2 allele (orange peaks) reads in the H3K27-Ac ChIP-seq data region of interest. 4 of these regions (arrows) are located in the vicinity of super-enhancers (indicated in blue). Non-informative (no SNP differences between FVB/N and 129P2) reads and reads without significant difference between FVB/N and 129P2 are not indicated

Suppl. figure 6

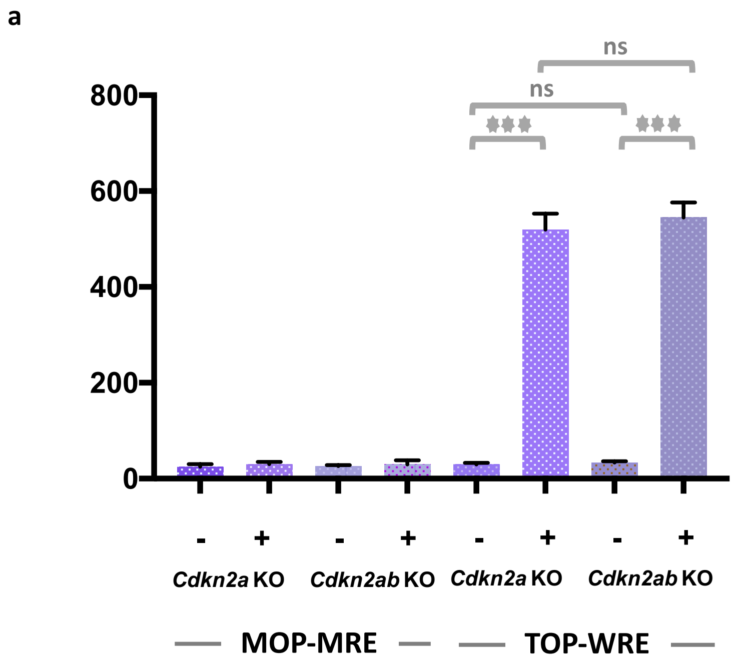

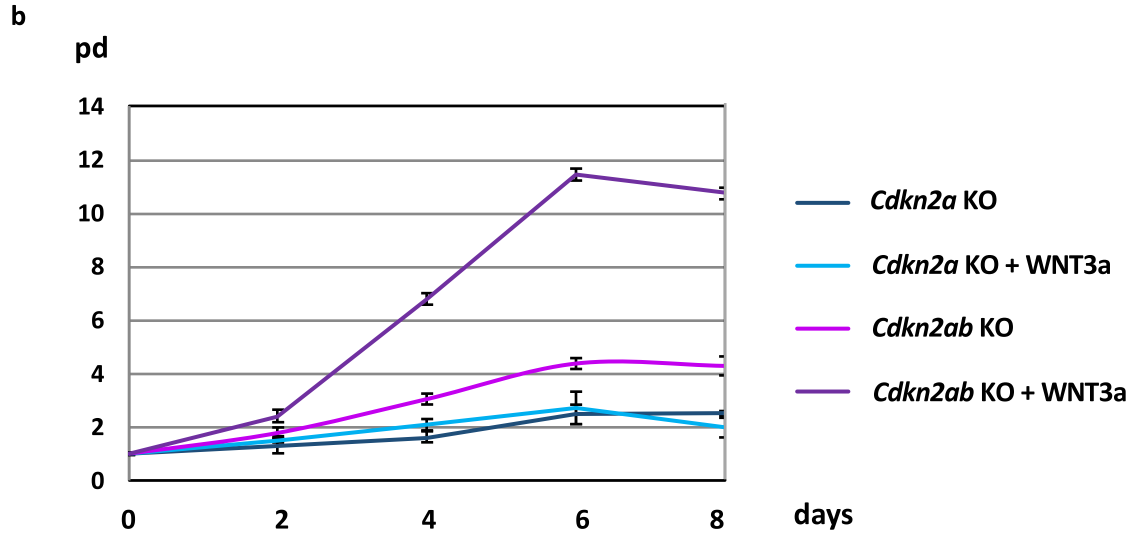

Suppl. figure 6) a, WNT induced β-catenin dependent transcription using a WNT responsive Luciferase reporter plasmid in *Cdkn2a* KO and *Cdkn2ab* KO MEFs with (+) and without (-) WNT3a supplementation. **b,** Proliferation of *Cdkn2a* KO and *Cdk2ab* KO MEFs in DMEM with or without WNT3a supplementation. Vertical axis: population doublings, horizontal axis: time in days.

Statistical analysis (Unpaired t test): ns: not significant, ***: p<0.001.

Suppl. figure 7

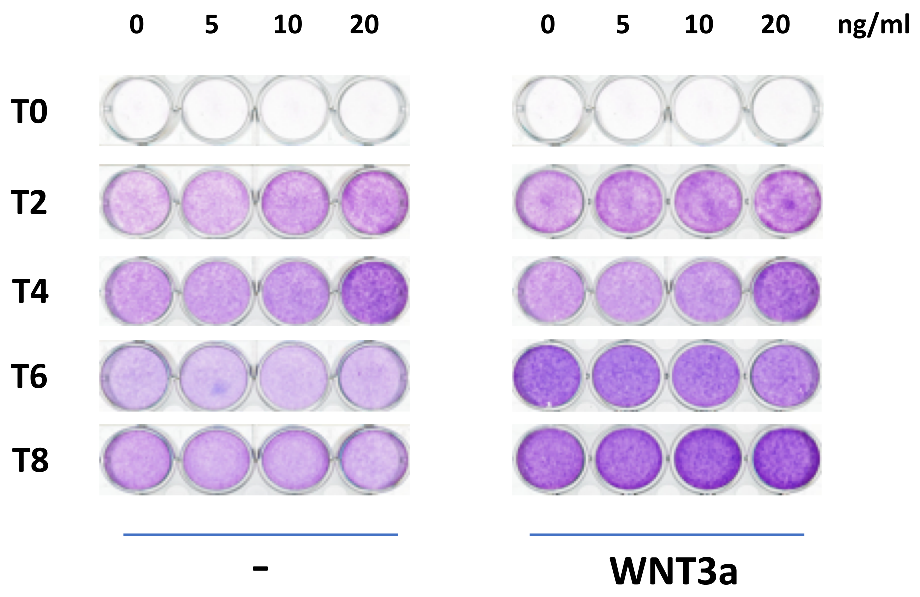

Suppl. figure 7) Proliferation under adherent conditions of *Cdkn2ab* KO MEFs in the presence of increasing concentrations of *Pdgfβ* with and without WNT3a supplementation.

Suppl. figure 8

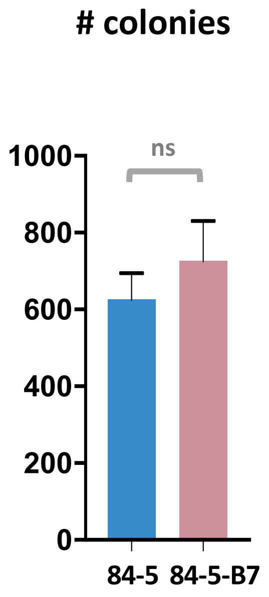

Suppl. figure 8) Anchorage independent colony formation of skin tumour cell line 84-5 and 84-5-P7 (*Pdgfβ* knockout).

Statistical analysis (Unpaired t test): ns: not significant.

Suppl. figure 9

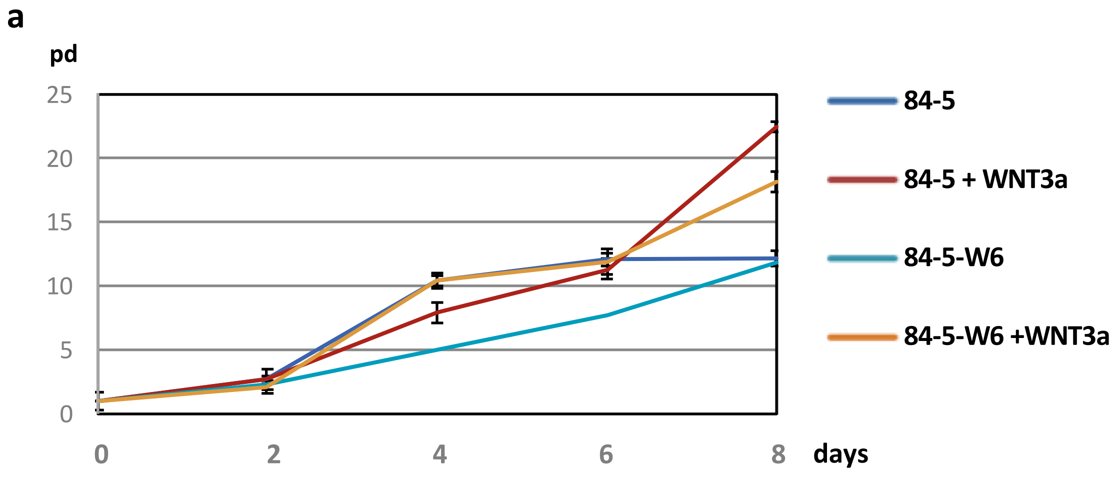

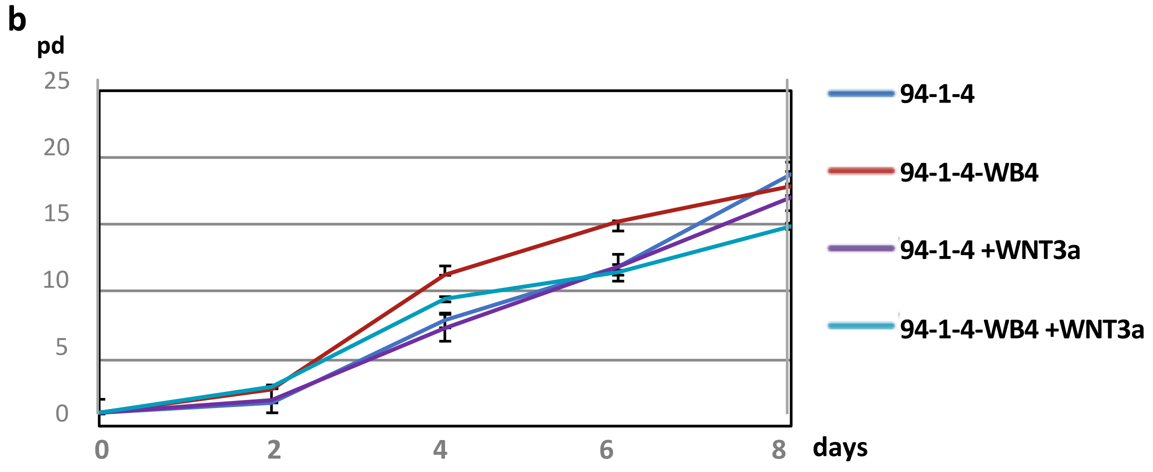

Suppl. figure 9) a, Proliferation of skin tumour cell lines 84-5 and 84-5-W6 (parental wt and *Wnt7b* KO derivative) in HITES medium with or without WNT3a supplementation. **b,** Proliferation of skin tumour cell lines 94-1-4 and 94-1-4-WB4 (parental wt and *Wnt7b* KO derivative) in HITES medium with or without WNT3a supplementation. Vertical axis: population doublings, horizontal axis: time in days.

Suppl. figure 10

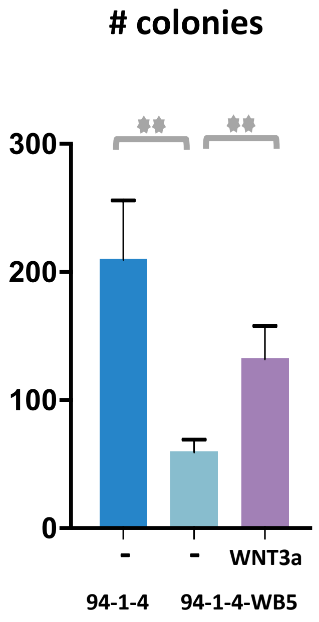

Suppl. figure 10) Anchorage independent colony formation of skin tumour cell line 94-1-4 and 94-1-4 WB5 (*Wnt7b* knockout) with and without WNT3a supplementation.

Statistical analysis: **: $p< 0.01$.

Suppl. Figure 11

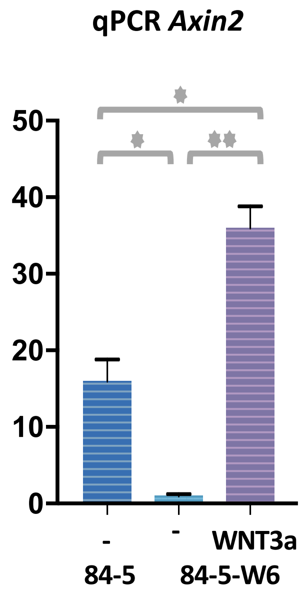

Suppl. Figure 11) mRNA analyses (qPCR) of *Axin2* relative to *Gapdh* of 84-5 and 84-5-W7 (*Wnt7b* knockout derivative) tumour cells with and without Wnt3a supplementation.

Statistical analysis (Unpaired t test): *: p< 0.05, **: p< 0.01.

Suppl. figure 12

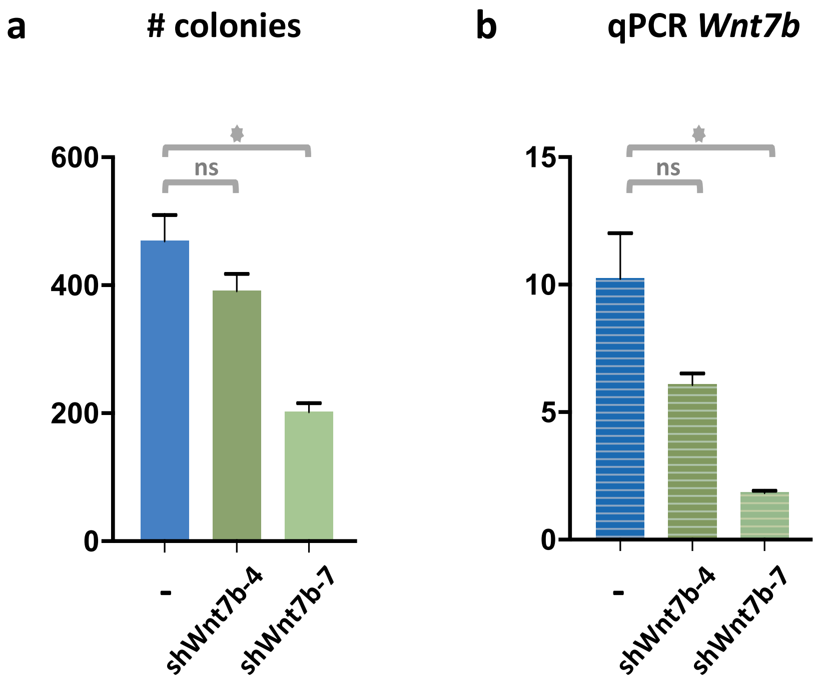

Suppl. figure 12) a, Anchorage independent colony formation of skin tumour cell line 84-5 and 84-5 cell populations expressing 2 independent *Wnt7b* short hairpin knockdown constructs 4 and 7. **b,** qPCR analysis on Wnt7b mRNA in skin tumour cell line 84-5 and 84-5 cell populations expressing 2 independent *Cdk6* short hairpin knockdown constructs 4 and 7.
Statistical analysis (Unpaired t test): ns: not significant, *: $p < 0.05$.

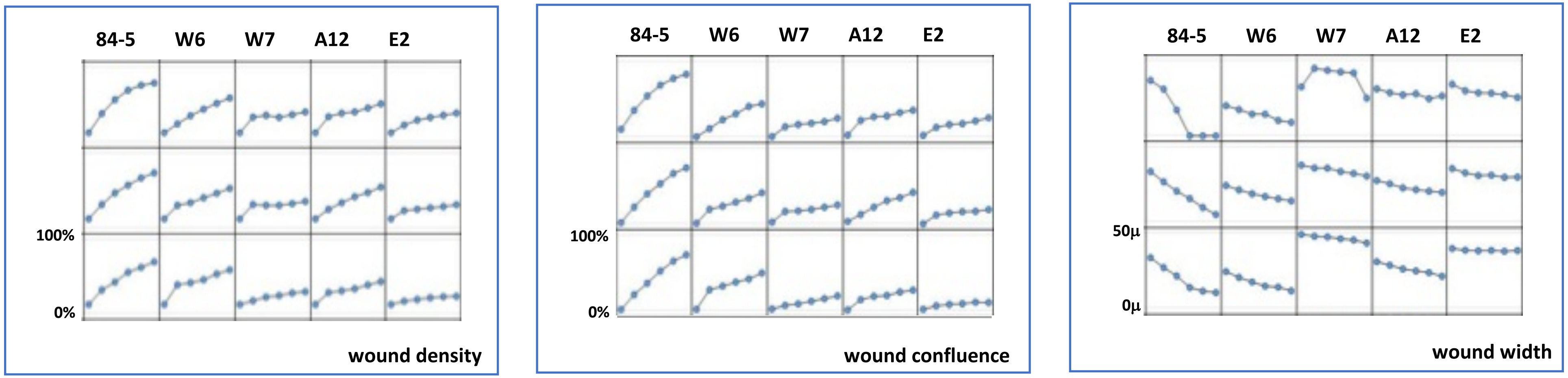

Suppl. figure 13) Migration of 84-5 tumour cells and gene edited derivatives: *Wnt7b* knockout: W6 and W7 and *Cdk6* knockout: A12 and E2; 0.5mm scratch wounds were applied to confluent monolayers and migration was followed over 20hrs. Migration is depicted as relative wound density (%, cell density in the wound area relative to the cell density outside the wound over time), wound confluence (%, cell confluence within the wound region over time) and width (µm, the distance between the wound boundaries over time).

Suppl. figure 14

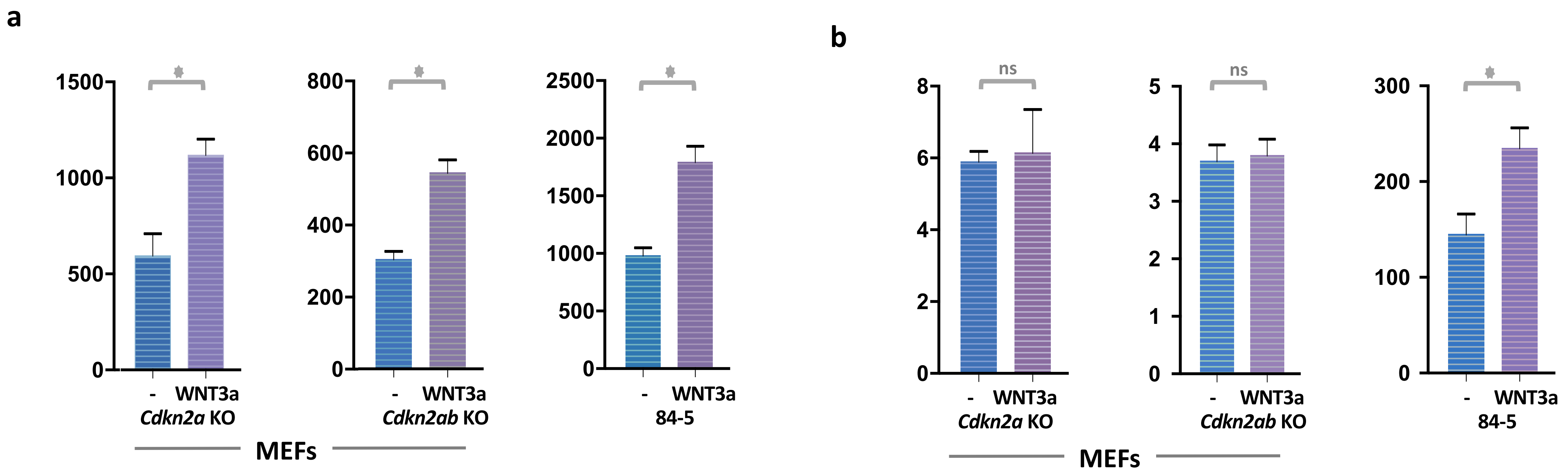

Suppl. figure 14) a, mRNA analyses (qPCR) of *Cyclin D1* relative to *Gapdh* of *Cdkn2a* KO MEFs, *Cdkn2a* KO MEFs and of 84-5 tumour cells with and without WNT3a supplementation. **b,** mRNA analyses (qPCR) of *Cdk6* relative to *Gapdh* of *Cdkn2a* KO MEFs, *Cdkn2a* KO MEFs and of 84-5 tumour cells with and without WNT3a supplementation.

Statistical analysis (Unpaired t test): ns: not significant, *: $p < 0.05$.

Suppl. figure 15

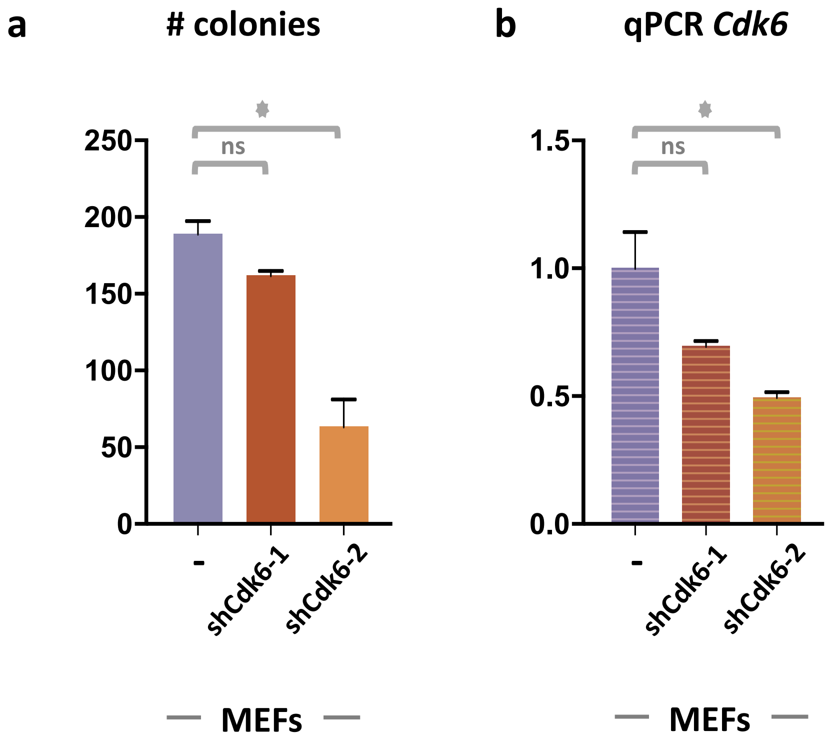

Suppl. figure 15) a, Anchorage independent colony formation of Wnt3a stimulated *Cdkn2ab*$^{-/-}$ MEFs expressing 2 independent *Cdk6* short hairpin knockdown constructs 1 and 2. **b,** Western blot analysis of Cdk6 protein expression in WNT3a stimulated *Cdkn2ab*$^{-/-}$ MEFs expressing 2 independent *Cdk6* short hairpin knockdown constructs 1 and 2.

Statistical analysis (Unpaired t test): ns: not significant, *: $p< 0.05$.

Suppl. figure 16

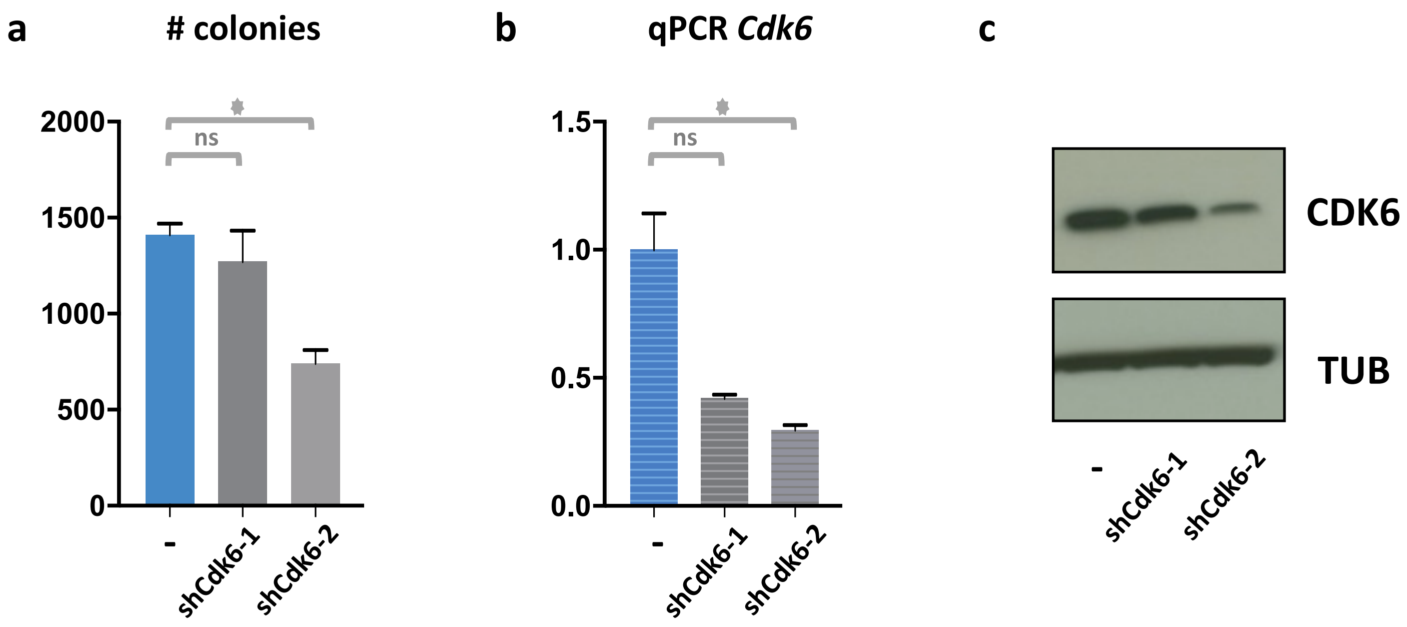

Suppl. figure 16) a, Anchorage independent colony formation of skin tumour cell lines 84-5 expressing 2 independent *Cdk6* short hairpin knockdown constructs 1 and 2. **b,** qPCR analysis on *Cdk6* mRNA in skin tumour cell line 84-5 and 84-5 cell populations expressing 2 independent *Cdk6* short hairpin knockdown constructs 1 and 2. **c)** Western blot analysis of Cdk6 protein expression in skin tumour cell line 84-5 and 84-5 cell populations expressing 2 independent *Cdk6* short hairpin knockdown constructs 1 and 2.

Statistical analysis (Unpaired t test): ns: not significant, *: $p < 0.05$.

Suppl. figure 17

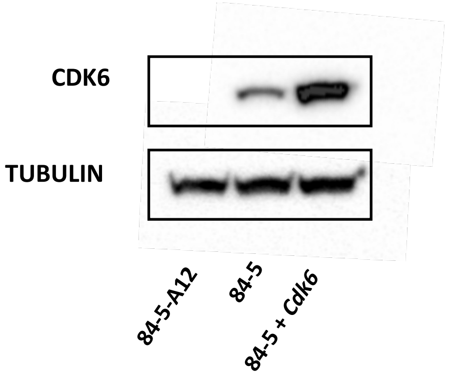

Suppl. Figure 17) Western blot analysis of CDK6 expression validating Cdk6 CRISPR/Cas inactivation and transduced CDK6 overexpression.

Supplementary table 1. Microsatellite markers used to identify the relevant 129P2 allele(s).

For sequence Mit markers see www.informatics.jax.org

D1Nki106	TGGTATGTCAAGTAGAGGCACTG	CAAGGGCCTTCGGAAAAA
D1Mit231		
D1Mit477		
D1Mit46		
D1Mit45		
D1Mit308		
D1Mit54		
D1Mit445		
D1Mit501		
D1Mit403		
D1Mit155		
D2Mit312		
D2Mit81		
D2Mit367		
D2Nki106	GACCAGTGCAAGCCAGCTAT	CTTGGAAGTCACCACAGTGC
D2Mit380		
D2Mit14		
D2Nki108	CAACTGCTACCTCAGAAGTATCTCA	GCTGCTTGTTAAACTCCCACT
D2Nki109	TCGTTATGTACACATGTGAATCTGTT	GCCTATTAATCATGAGGCTTCC
D2Mit26		
D2Mit148		
D3Mit60		
D3Mit367		
D3Mit178		
D3Mit239		
D3Mit21		
D3Mit6		
D3Mit241		
D3Mit49		
D3Mit103		
D3Mit319		
D3Mit113		
D3Nki108	TGTCCTCCATATGGGCAAAG	CATGGCGTAACGAAACAAAA
D4Mit149		
D4Mit264		
D4Mit1		
D4Mit108		

D4Mit82		
D4Mit152		
D4Mit37		
D4Mit68		
D4Mit148		
D4Mit190		
D5Mit145		
D5Mit176		
D5Mit11		
D5Mit304		
D5Mit188		
D5Mit321		
D16Mit197		
D6Mit166		
D6Mit160		
D6Mit123		
D6Mit8		
D6Mit55		
D6Mit15		
D7Nki134	GATCTGATCTGACGCCCATT	TTGTCTTCCGAGCTCTACACA
D7Nki135	TCATGAAATGAAGGGTGTGC	AGCTGTCGCCTGACTTTACG
D7Nki137	GGCAGCAGAAACAGGAGTTC	CCCAACCATTGGGTTGTTTA
D7Mit316		
D7Nds2		
D7Mit31		
D7Mit66		
D7Nki118	TCTGGCCTCCTTAGGTGCTA	TCATTCCATGAACCAGCTTTC
D8Nki114	CATCAGCTCTGCATCTGACA	TCTCTTTAGATCTGTTCCCCACTT
D8Mit125		
D8Nki112	CCAAACAAGACTGACCCTGTC	GGGGAAGCTGAAAAATGGAT
D8Mit40		
D8Mit56		
D9Nki106	TACCCAGCCTTCAAGGTAGC	ATGTGTGTATCCACCCAGCA
D9Mit64		
D9Mit227		
D9Mit32		
D9Mit9		
D9Mit168		

D10Mit1		
D10Mit87		
D10Mit38		
D10Mit129		
D10Mit230		
D10Mit133		
D10Nki106	AAATCAGGGAGGCAAAACCT	TTTCTTCAAATTCTTTCTCCAATGT
D11Mit71		
D11Mit53		
D11Mit164		
D11Mit262		
D11Mit67		
D11Mit338		
D12Nki101	CCCAGCCATTGAGCTGATTA	CAGTGTTGGCAGCACAAGTT
D12Mit85		
D12Mit147		
D12Mit35		
D12Mit51		
D12Mit7		
D12Mit134		
D13Mit162		
D13Mit17		
D13Mit21		
D13Mit233		
D13Nki103	GGAAGACATGCAAGCAACAA	CGCAGAGACATACCATTGAGA
D14Mit1		
D14Mit15		
D14Mit37		
D14Mit39		
D14Mit94		
D14Nki107	AACAAAAACATCCAGGATAGCTG	TGGAATTCCTTGAGCCCTAA
D15Nki104	GGACTGACTCCTGTGGTTGTC	TGGTGCAAGCCTTCCTAAGT
D15Nki102	GGCACCACCCACAGTAAATC	ATAGGGGATTTTCGGACGAG
D15Mit5		
D15Nki103	GCCCAGTAAGATCACAAGCA	GGGGGTAAGGCTTTGTTTTC
D15Nki113	GAAGTTGGGAGCCACACATT	CCCGACCTTGTCACTTTTCT
D15Mit107		

D15Mit35		
D16Mit32		
D16Nki105	TGCACAGTTTTAATTCCATTGC	GGCCAACAAACACTCAAAGG
D16Mit42		
D16Mit139		
D16Nki106	TCCTATTTCCTTGGCAACCA	TTCAATACTCCCTTGGCATTG
D17Nki106	CGAGAATTCTGTCTTGGTATAGTTG	ACTGTTTTAAGAAAATCTGGAAGG
D17Mit46		
D17Mit115		
D17Mit93		
D17Nki114	AGCCTGGGCTACCTGGAC	TTCCTATCTCCTGGCTGCTG
D18Mit19		
D18Mit68		
D18Mit17		
D18Mit123		
D18Mit49		
D18Nki104	GCATTAGATGCTTCCTTTGTCC	TCCTTGCATGTTTCAGGTCA
D19Mit68		
D19Mit106		
D19Mit3		
D19Mit33		

134 markers

Fine mapping:

D15Mit232		
D15Nki126	AATTTGTGAGTGCGTGTGGA	AGCAAGAATGTGCCACCAC
D15Nki168	CCTCTGTGACCCAAGTCCAT	ACCCTTCACAGAGAGCCAGT
D15Nki113	GAAGTTGGGAGCCACACATT	CCCGACCTTGTCACTTTTCT
D15Nki117	AGCAACCACTAACTGCACTGT	TGCTCTAGAGGAACAGAAAGCA
D15Nki145	TCCATTTTCCTGCAGTTTTT	TCAACCTAACTGCCCACTGA
D15Nki147	TGGGGAAGTTTTTGAGTTTG	CAGAGCCCATCACATCACAC
D15Nki148	CCTCTTTGCATGCTGTTAAGG	TGGTGAGATCCTCAAACGTACA

8 markers

Supplementary table 2: list of genes on relevant region on chr 15.

List of genes in the Chr15: 67-87Mb region. Relevant SNP markers and the Super Enhancer are also indicated. Genes in the smallest region identified from 129P2 background are proceeded by a blue coloured cell and genes in the crossover region are proceeded by a grey cell. Genes reported to be involved in carcinogenesis are indicated in bold in a purple cell. Markers are indicated in a yellow cell and the super enhancer in red in a green cell.

	D15Nki126	
	St3gal1	ST3 beta-galactoside alpha-2,3-sialyltransferase 1
	Zfat1	zinc finger and AT-hook domain containing
	Khdrbs3	KH RNA binding domain containing, signal transduction associated 3
	Col22a1	collagen type XXII alpha 1 chain
	Kcnk9	potassium two pore domain channel subfamily K member
	1810044A24Rik	
	Chrac1	chromatin accessibility complex subunit 1
	Eif2c2	argonaute 2, RISC catalytic component
	Ptk2	protein tyrosine kinase
	Dennd3	DENN domain containing
	Scl45a4	
	Gpr20	G protein-coupled receptor
	pPtp4a3	protein tyrosine phosphatase
	Gm628	maestro heat-like repeat family member 5
	Bai1	brain-specific angiogenesis inhibitor 1
	Arc	activity regulated cytoskeleton associated protein
	Jrk	helix-turn-helix protein
	Psca	prostate stem cell antigen
	4930572J05Rik	
	Slurp1	secreted LY6/PLAUR domain containing 1
	Lypd2	LY6/PLAUR domain containing 2
	Lynx1	Ly6/neurotoxin 1
	Ly6d	lymphocyte antigen 6 family member
	Ly6k	lymphocyte antigen 6 family member
	Gml	glycosylphosphatidylinositol anchored molecule like
	Cyp11b1	cytochrome P450 family 11 subfamily B member
	Cyp11b2	cytochrome P450 family 11 subfamily B member
	Ly6e	lymphocyte antigen 6 family member
	Ly6h	lymphocyte antigen 6 family member
	Gpihbp1	glycosylphosphatidylinositol anchored high density lipoprotein binding protein
	Zfp41	zinc finger protein
	Top1mt	DNA topoisomerase I mitochondrial
	Rhpn1	rhophilin Rho GTPase binding protein

Mafa	bZIP transcription factor A
**Gsdmdc1**	gasdermin D, epithelial proliferation
Naprt1	nicotinate phosphoribosyltransferase
Eef1d	eukaryotic translation elongation factor 1 delta
Tigd5	tigger transposable element derived
Pycrl	pyrroline-5-carboxylate reductase-like
**Tsta3**	tissue specific transplantation antigen
Zfp623	zinc finger protein
Zfp707	zinc finger protein
**Mapk15**	mitogen-activated protein kinase
AA409316	
**Scrib**	scribbled planar cell polarity protein, tumor suppression
2410104IRik	
Eppk1	epiplakin, integrity of keratin intermediate filament network
Plec1	Plectin, cytoskeleton
LOC671535	
Grina	glutamate ionotropic receptor
Spatc1	spermatogenesis and centriole associated 1
LOC100041269	
Oplah	5-oxoprolinase, ATP-hydrolysing
Exosc4	exosome component
Gpaa	G-protein subunit alpha
Cyc1	cytochrome c1
Sharpin	SHANK associated RH domain interactor, regulation of integrin or NF-κB activity
Maf1	MAF1 homolog, negative regulator of RNA polymerase III
Brp16	brain protein
Bop1	block of proliferation, ribosome biogenesis
Scx	scleraxis bHLH transcription factor
Hsf1	heat shock transcription factor
Dgat1	diacylglycerol O-acyltransferase, metabolism
Scrt1	scratch family transcriptional repressor 1
Fbxl6	F-box and leucine rich repeat protein
Gpr172b	solute carrier family 52 member 1, vitamin B2 transporter
Adck5	aarF domain containing kinase 5
Cpsf1	cleavage and polyadenylation
Scl39a4	
Vps28	ESCRT-I subunit
**Nfkbil2**	tonsoku like, DNA repair protein
Cyhr1	cysteine and histidine rich 1
Kifc2	kinesin family member C2
**Foxh1**	forkhead box H1
Ppp1r16a	protein phosphatase 1 regulatory subunit
Gpt1	glucose 6-phosphate/phosphate translocator

Mfsd3	major facilitator superfamily domain containing
**Recql4**	RecQ like helicase, various syndromes and cancers(?)
Lrrc14	leucine rich repeat
Lrrc24	leucine rich repeat
C030006K11Rik	
D15Wsu169e	
Zfp251	zinc finger protein
Zfp7	zinc finger protein
Commd5	COMM domain containing 5
Zfp647	zinc finger protein
1110038F14Rik	
Mb	
Apol6	apolipoprotein L6
Rbm9	RNA binding protein
Apol2	apolipoprotein L2
Myh9	myosin heavy chain 9
Txn2	thioredoxin 2, mitochondrial membrane potential, anti oxidant-induced apoptosis
Foxred2	FAD dependent oxidoreductase
Eif3s7	translation initiation factor
Cacng2	calcium voltage-gated channel
Rabl4	intraflagellar transport
Pvalb	parvalbumin, neuronal
Ncf4	neutrophil cytosolic factor 4, auto-immunity?
Csf2rb2	colony stimulating factor 2 receptor, beta 2
1700061J05Rik	
Tst	thiosulfate sulfurtransferase
Mpst	mercaptopyruvate sulfurtransferase
Kctd17	potassium channel tetramerization
Tmprss6	transmembrane serine protease, matrix remodelling
Il2rb	interleukin 2 receptor subunit beta
Sstr3	somatostatin receptor 3
Rac2	Rac family small GTPase 2, ras signalling various cancers
Pscd4	cytohesin 4
Lrrc62	extracellular leucine rich repeat and fibronectin type III domain
Mfng	O-fucosylpeptide 3-beta-N-acetylglucosaminyltransferase
Card10	caspase recruitment
Cdc42ep1	CDC42 binding protein, actin polymerization
Lgals2	beta-galactoside binding lectin
Gga1	golgi associated, gamma adaptin ear containing, ARF binding protein 1
Sh3bp1	Rho GTPase activating protein
Pdxp	pyridoxal phosphatase, vitamin b6 metabolism
Lgals1	beta-galactoside-binding protein, cell-cell-matrix interaction
Nol12	RNA binding protein

Triobp	trio and F-actin binding protein,nonsyndromic deafness
H1f0	H1 histone family member
Gcat	glycine C-acetyltransferase
Galr3	galanin receptor
Ankrd54	ankyrin repeat domain
Eif3s6ip	translation initiation factor 3
1700088E04Rik	
Polr2f	subunit of RNA polymerase II
Sox10	SRY-related HMG-box transcription factor, neural crest, peripheral nervous system
Pick1	protein interacting with PRKCA 1
Scl16a8	monocarboxylate transporter macular degeneration
Baiap212	
Pla2g6	A2 phospholipase neuronal
Maff	leucine zipper (bZIP)-type transcription factors
4732495E13Rik	
Csnk1e	casein kinase I
Kcnj4	potassium voltage-gated channel
Kdelr3	endoplasmic reticulum protein retention receptor
Ddx17	RNA helicase
Dmc1	meiotic recombination
4933432B09Rik	
**Pgea1**	β-catenin antagonist, myeloid leukemia?
Tomm22	mitochondrial import receptor
Gtpbp1	
Unc84b	nuclear membrane
**Dnalc4**	possible involvement myeloid leukemia
Nptxr	Neuronal
Cbx6	Polycomb Repressive Complex 1
Apobec3	Many cancers
Cbx7	Polycomb Repressive Complex 1
D15Nki168	
**Pdgfb**	Many cancers
Syngr1	risk of rheumatoid arthritis and primary biliary cirrhosis
**Map3k7ip1**	Tgf beta signaling
Mgat3	N-glycosylation of plasma proteins
Smcr7l	mitochondrial dynamics
Atf4	response to a diverse array of microenvironmental stresses
Rps19bp1	ribosomal protein
Cacna1i	Voltage-gated calcium channel
Enthd1	
Grap2	adaptor protein specifically expressed in lymphoid tissues
AW544981	

Tnrc6b	involved in miRNA-mediated mRNA degradation
Adsl	Adenylosuccinate lyase, de novo purine synthesis
Rutbc3	
Mkl1	Leukaemia coactivator serum responds factor
Mchr1	Melanin receptor
**Slc25a17**	Neuroblastoma?
**St13**	Suppressor proliferation
Dnajb7	
**Ep300**	Bromo-domain, involved in many cancers
L3mbtl2	Polycomb
Rangap1	Atp-ase, myeloma, glioma
Zc3h7b	Endometrial sarcoma
Tef	
**Tob2**	candidate tumor suppressor to transcriptionally repress proto-oncogene cyclin D1
Phf5a	PHD-finger protein 5a maintaining pluripotency
Aco2	Mitochondrial metabolic enzyme
Polr3h	RNA polymerase III subunit
Csdc2	cold shock domain containing C2
Pmm1	phosphomannomutase
D15Wsu75e	
**Xrcc6**	double strand break repair
4930407I10Rik	
Mei1	meiotic recombination
Ccdc134	an immune cytokine, cell proliferation through the JAK3-STAT5 pathway
Srebf2	tumor suppressive, increases cholesterol and lipid levels
LOC435145	
**Tnfrsf13c**	Baffr, leukemia
Cenpm	centromere protein M
Sept	subfamily of GTPases, cytokinesis
Wbp2nl	Breast cancer
Naga+B53	α-*N*acetylgalactosaminidase cisplatin drug response
C920005C14Rik	
1500032L24Rik	
Ndufa6	Mitochondrial complex I
Cyp2d22	cytochrome P450
Tcf20	transcription factor 20
**Nfam1**	NFAT activating protein, various cancers?
Serhl	Lnc-RNA
Poldip3	regulates DNA replication and mRNA translation
Cyb5r3	Metabolism
A4galt	role of glycosphingolipids in the maintenance of epithelial cancer
Arfgap3	transport of cation-independent mannose 6-phosphate receptor
Pacsin2	protein kinase C and casein kinase, neuronal

Ttll1	tubulin tyrosine ligase like 1
Bik	Apoptosis induction
Mcat	suppresses tumor progression
Tspo	steroidogenesis and apoptosis
Ttll12	Tubulin, marker
Scube1	Boomerker breast renal cancer
Mpped1	metallophosphoesterase
4931407K02Rik	
Sult4a1	sulfotransferase family 4A
Pnpla5	Liver pathology
Pnpla3	Liver pathology
Samm50	mitochondrial
Parvb	parvin beta, actin-binding
Parvg	parvin gamma, actin-binding
1810041L15Rik	
**Ldoc1l**	regulator of NFKB signaling
Arhgap8	Breast colon cancer rho gpt-ase
3110043J09Rik	
**Phf21b**	Tumor suppressor vs oncogene prostate
Nup50	Nucleo pore complex
5031439G07Rik	
**Upk3a**	Bladder cancer marker
3110048E14Rik	
Smc1b	Cohesin
Ribc2	RIB43A domain with coiled-coils 2
Fbln1	ECM
Atxn10	Ataxin 10, spinocerebellar
**Wnt7b**	wnt ligand
**Sup. Enh.**	
**Let7b, Let7c**	miRNA
D15Nki117	
Ppara	Liver
2210021J22Rik	
Pkdrej	
AW124722	
Gtse1	Microtubel breast kidney
Trmu	
Celsr1	GBM kidney
BC021523	
Cerk	
**Tbc1d22a**	TBC1 domain family member, HCC?
AW049604	
D15Nki147	

Supplemenrary table 3: Primer sequences

Primer sequences used in RNA analysis by qPCR, construction of shRNA expression constructs and gRNA expression constructs

RNA expression analysis by qPCR

Wnt7b	exon 3 frw:	TCGACTTTTCTCGTCGCTTT
	exon 4 rev:	AGGCTTCTGGTAGCTGCGTA
Pdgfβ	exon 5 frw:	TGGGACATCCAGGGAGCA
	exon 6 rev:	CCTTGTCATGGGTGTGCTTA
Fbln1	exon 7 frw:	AGCTGTGGGACTGGCTATG
	exon 9 rev:	TGCTCTTGGGATGGCTG
Axin2	exon 5 frw:	CAAGACCAAGGAGGAGATCG
	exon 6 rev:	TTTTGGCAAGGTACCACCTC
Cdk6	exon 7 frw:	CTCAACCCATCGAGAAGTTTG
	exon 8 rev:	GTTGGATGGCAGGTGAGAGT
CyclinD1	exon 3 frw:	TGGTGAACAAGCTCAAGTGG
	exon4 rev:	GCAGGAGAGGAAGTTGTTGG
Gapdh	exon 4 frw:	GCCGGCTCATCACACAGT
	exon 4 rev:	TCGGAGTCCTCAGTCTCACTC
Actin	exon 4 frw:	CACAACGTGCCCATCTATGA
	exon 5 rev:	GGCCATCTCATTCTCGAAGT

Allele specific RNA expression analysis

Wnt7b	exon 4 frw:	AACACGCACCAGTACACCAA
	exon 4 rev:	GCTCCAGAAGCAAAGAAGGA
Pdgfβ	exon 5 frw:	TGGGACATCCAGGGAGCA
	exon 7 rev:	TCGGGCTAAGTGCCAGGCT
Fbln1	exon 15 frw:	AGGAGGGCTTTTTCACCACT
	exon 15 rev:	TGCTCTTGGGATGGCTG

Guide RNA expression by pX330 vectors

Wnt7b		
exon 3 excision:	gRNA1:	GGTGCAATCCACTTGCTGTA
	gRNA2:	CGGGCAGAAAGGTAGCCGTT
Pdgfβ		
exon 2 excision:	gRNA1:	TCCTGGTGTCTGACCGACAG
	gRNA2:	AGGAGGGTCCGATTTACCTA
Cdk6		
exon3 mutation:	gRNA1:	GCCCGCGACCTGAAGAACGG
	gRNA2:	GACCTTCGAGCACCCCAACG

ShRNA expression by lentiviral knockdown constructs (pLKO.1 vector)

Wnt7b	Wnt7b-4:	GCTACCTAAGTTCCGCGAGGT
	Wnt7b-7:	CGGAGCATTGTCATCCGTGGT
Cdk6	Cdk6-1:	CGGTTGCATCTTTGCAGAAA
	Cdk6-2:	ATCTTCTAGAGATAACTACTT

Supplementary table 4

List of antibody used in this study

Histochemistry

Antibody	Dilution	Supplier/Cat. No.
β-Catenin	1:200	Abcam/ab32572
CK 5	1:500	Covance Babco/PRB-160P
CK 8	1:600	University of Iowa/Troma-1
CK 10	1:500	Covance/Babco/PRB-159P
CK 14	1:6000	ABCAM/ab181595
LEF1	1:100	Cell signaling/2230
P-Cadherin	1:100	R&D systems/AF761
SOX9	1:3500	Millipore/AB5535
WNT7B	1:100	Abcam/ab94915
SOX2	1:200	Cell signaling/149625
P63	1:600	BD Pharmigen/559951

Western blot analysis

Antibody	Dilution	Supplier/Cat. No.
vinculin	1:10.000	Sigma V9131, mouse
Wnt7b	1:500	Abcam AB94915 rabbit
Myc-tag	1:500	Covance MMS-150R mouse
Cdk6	1:1000	AHZ0232 mouse
β-tubulin	1:5000	Abcam AB6046 rabbit
Actin	1:5000	Abcam AB8224 mouse

Chipseq

Antibody		Supplier/Cat. No.
H3k27Ac		Abcam ab4729
